# Adoption of single agent anticancer therapy for advanced hepatocellular carcinoma and impact of facility type, insurance status, and income on survival: Analysis of the national cancer database 2004–2014

**DOI:** 10.1002/cam4.3985

**Published:** 2021-05-31

**Authors:** Aman Opneja, Gino Cioffi, Asrar Alahmadi, Nelroy Jones, Tin‐Yun Tang, Nirav Patil, David L. Bajor, Joel N. Saltzman, Amr Mohamed, Eva Selfridge, Ankit Mangla, Jill Barnholtz‐Sloan, Richard T. Lee

**Affiliations:** ^1^ University Hospitals Cleveland Medical Center Cleveland OH USA; ^2^ Department of Population and Quantitative Health Sciences Case Western Reserve University School of Medicine Cleveland OH USA; ^3^ Case Western Reserve University Comprehensive Cancer Center Cleveland OH USA

**Keywords:** chemotherapy, facility type, hepatocellular carcinoma, income, insurance, survival

## Abstract

**Background:**

This study analyzes the pattern of use of single agent anticancer therapy (SAACT) in the treatment and survival of advanced hepatocellular carcinoma (aHCC) before and after sorafenib was FDA approved in 2007.

**Methods:**

Adult patients diagnosed with HCC and treated with only ACT from 2004 – 2014 were identified in NCDB database. Patients were analyzed during three time frames: 2004–2006 (pre‐sorafenib (PS)), 2007–2010 (early sorafenib (ES)) and 2011–2014 (late sorafenib (LS)). Cox proportional hazards models and Kaplan‐Meier method were used for analyses.

**Results:**

The NCDB contained 31,107 patients with HCC diagnosed from 2004–2014 and treated with ACT alone. Patients were generally men (78.0%), >50 years of age (92.5%). A significant increase in the rate of adaption of SAACT was observed over time: 6.2% PS, 15.2% ES, and 22.2% LS (*p* < 0.0001). During this later period, the highest proportion of SAACT is among academic and integrated network facilities (23.3%) as compared to community facilities (17.0%, *p* < 0.0001). The median overall survival of patients with aHCC treated only with SAACT improved significantly over time from 8.0 months (m) (95% CI: 7.4–8.8) to 10.7 m (10.4–11.2) to 15.6 m (15.2–16.0, *p* < 0.001). Multivariate analysis indicates worse outcomes for patients treated at community cancer programs (HR 1.28, (5% CI: 1.23–1.32), patients without insurance (HR 1.11, 1.06–1.16) and estimated household income of <$63,000 (HR 1.09, 1.05–1.13).

**Conclusion:**

aHCC patients treated only with ACT have experienced an overall improvement in survival, but significant differences exist between facility type, insurance status, and income.

## 1 INTRODUCTION

Hepatocellular carcinoma (HCC) is the fifth most commonly diagnosed cancer in adult men and the ninth most common cancer in women, and represents the fourth leading cause of cancer‐related death worldwide.^1^ Although the incidence of HCC is on decline in certain countries in Asia with increased vaccination for hepatitis B, it has been increasing in other counties including the United States.^2^, ^3^ Globally, hepatitis B is the most common etiology of HCC but in the US chronic hepatitis C infection remains a major cause of HCC.^4^, ^5^ Other common etiologies include alcohol‐related liver cirrhosis, nonalcoholic fatty liver, and nonalcoholic steatohepatitis.^6^ Various factors affect survival in patients with HCC. Previous studies have identified elevated AFP, Child‐Pugh stage, female gender, ascites, and multifocal disease as prognostic factors affecting survival.^7^, ^8^ Overall prognosis of HCC is poor with a 5‐year survival of less than 20% making it one of the fastest growing causes of cancer‐related death in the US.^9^

Treatment options for early stage HCC include surgical resection, liver transplantation, or locoregional therapies such as radiofrequency ablation. Prior to 2007, patients with advanced HCC (aHCC) were treated with transarterial embolization and radioembolization with limited long‐term control. Sorafenib was the first targeted agent for aHCC to get FDA approval in November 2007. Sorafenib is an oral multi kinase inhibitor that inhibits both intracellular and surface kinases which are involved in tumor signaling. Inhibition of these kinases affect tumor proliferation, angiogenesis and cell death.^10^ Its approval was based on a large phase 3 multicenter clinical trial, which showed a significant improvement in overall survival (10.7 vs 7.9 months (m)) as compared to placebo.^11^ Sorafenib remained the main treatment option for aHCC for approximately a decade as the next FDA approval in aHCC was regorafenib in 2017.

Since the approval of sorafenib, limited data exist regarding its pattern of adoption and socioeconomic factors that may affect outcomes among the general aHCC patient population in the United Sates.^12^ Because sorafenib was the only approved systemic therapy for a decade, we are able to examine the impact of this therapy for aHCC through the National Cancer Database (NCDB). In this study, we analyzed trends of adoption of single agent anticancer therapy (SAACT) among different facility types and its impact on survival as well as socioeconomic factors in this patient population. This research can help provide insight into real world outcomes and analyze factors that affect outcomes in the general population.

## 2 METHODS

The NCDB is jointly sponsored by the American College of Surgeons and the American Cancer Society and is a clinical oncology database sourced from hospital registry data collected from more than 1,500 Commission on Cancer accredited facilities. We received approval from the NCDB to conduct this study and obtained a dataset for HCC from 2004–2014. Using NCDB Participant User File, patients ≥18 years with diagnosis of hepatocellular carcinoma were identified by ICD code. We submitted our research protocol to the IRB for review and it was determined that approval was not required.

Data variables including age, sex, co‐morbidity index (Charlson/Deyo score), treatment type (single agent chemotherapy, combination chemotherapy), time of treatment, AFP (elevated or normal), facility type (academic, integrated network cancer centers, comprehensive community centers, and community centers), insurance type (Medicare, private, other, none) and estimated household income (<38 k, 38–47 k, 48–62 k, and >63 k) were obtained. Of note, systemic anticancer therapies (ACT) are categorized as chemotherapy in NCDB, and it does not differentiate between conventional chemotherapy (ex. fluorouracil) and targeted therapies such as sorafenib. For facility type, the academic and integrative network categories were combined as many academic centers are categorized as integrative network if they include multiple sites. When assessing for ACT and SAACT utilization, we excluded all patients that received any type of surgery, locoregional therapy, or radiation treatment. In general, we assessed outcomes according to three time frames: 2004–2006 (pre‐sorafenib), 2007–2010(early sorafenib) and 2011–2014 (late sorafenib). Cox proportional hazards models were used for univariate and multivariable analyses. Kaplan‐Meier method was used for survival analysis. Patients diagnosed in 2015 were excluded from the database due to insufficient data. SAS version 9.4 was used to perform all analyses, and R version 3.6.3 was used to generate all figures.

## 3 RESULTS

A total of 31,107 patients were identified in the NCDB database who were diagnosed with HCC and received treatment from 2004 – 2014. The majority were men (78.0%), White (73.0%), and between the ages of 50–69 years (66.0%). Other racial groups included Black (16.1%), Asian and Pacific Islanders (7.4%), and American Indian (0.8%). Nearly two‐thirds of patients (63.9%) were noted to have an elevated AFP, and 76.0% had a Charlson Deyo score of 0–1. Approximately three‐fourths of patients (72.9%) were estimated to have a household median income <$63,000. Almost all patients had insurance including Medicare (43.4%), private insurance (32.9%), and other insurance (17.0%). About five percent were noted to have no insurance. Patients were treated at three types of facilities: academic/integrated cancer centers (69.3%), comprehensive community centers (25.5%), and community centers (4.0%). (Table 1).

**TABLE 1:** Patient demographics

	Overall	2004–2006	2007–2010	2011–2014
Patients (*n*, %)	31,107 (100%)	3,701 (11.9%)	10,876 (35.0%)	16,530 (53.1%)
	Age at Diagnosis (*n*, %)			
<50 years	2,348 (7.5%)	502 (13.6%)	926 (8.5%)	920 (5.6%)
50–69 years	20,530 (66.0%)	2,111 (57.0%)	7,007 (64.4%)	11,412 (69.0%)
>70 years	8,229 (26.5%)	1,088 (29.4%)	2,943 (27.1%)	4,198 (25.4%)
	Sex (n, %)			
Male	24,010 (78.0%)	5,035 (78.0%)	8,459 (78.0%)	10,516 (77.9%)
Female	6,784 (22.0%)	1,417 (22.0%)	2,381 (22.0%)	2,986 (22.1%)
	Race (*n*, %)			
White	22,696 (73.0%)	2,660 (71.9%)	7,877 (72.4%)	12,159 (73.6%)
Black	5,005 (16.1%)	563 (15.2%)	1,703 (15.7%)	2,739 (16.6%)
Asian/Pacific Islander	2,293 (7.4%)	22 (0.6%)	93 (0.9%)	132 (0.8%)
American Indian	247 (0.8%)	369 (10.0%)	867 (8.0%)	1,057 (6.4%)
Unknown	866 (2.8%)	87 (2.4%)	336 (3.1%)	443 (2.7%)
	Primary Tumor Stage (*n* *n*, %)			
1	8,535 (27.4%)	593 (16.0%)	2,683 (24.7%)	5,259 (31.8%)
2	6,574 (21.1%)	554 (15.0%)	2,349 (21.6%)	3,671 (22.2%)
3	7,794 (25.1%)	1,161 (31.4%)	3,008 (27.7%)	3,625 (21.9%)
4	4,650 (14.9%)	531 (14.3%)	1,484 (13.6%)	2,635 (15.9%)
Unknown	3,554 (11.4%)	862 (23.3%)	1,352 (12.4%)	1,340 (8.1%)
	Alpha fetoprotein (*n*, %)			
Negative/Normal	5,599 (18.0%)	2,246 (60.7%)	6,920 (63.6%)	10,702 (64.7%)
Positive/Elevated	19,868 (63.9%)	507 (13.7%)	1,822 (16.8%)	3,270 (19.8%)
Unknown	5,640 (18.1%)	948 (25.6%)	2,134 (19.6%)	2,558 (15.5%)
	Charlson/Deyo score (*n*, %)			
0	14,916 (48.0%)	1,968 (53.2%)	5,135 (47.2%)	7,813 (47.3%)
1	8,719 (28.0%)	1,039 (28.1%)	3,079 (28.3%)	4,601 (27.8%)
2	3,374 (10.8%)	346 (9.3%)	1,239 (11.4%)	1,789 (10.8%)
>=3	4,098 (13.2%)	348 (9.4%)	1,423 (13.1%)	2,327 (14.1%)
	Facility type (*n*, %)			
Academic & Integrated Network	21,547 (69.3%)	2,535 (68.5%)	7,418 (68.2%)	11,594 (70.1%)
Comprehensive community	7,924 (25.5%)	912 (24.6%)	2,859 (26.3%)	4,153 (25.1%)
Community	1,235 (4.0%)	153 (4.1%)	446 (4.1%)	636 (3.8%)
Unknown	401 (1.3%)	101 (2.7%)	153 (1.4%)	147 (0.9%)
	Insurance status (*n*, %)			
Medicare	13,485 (43.4%)	1,586 (42.9%)	4,615 (42.4%)	7,284 (44.1%)
Private insurance	10,241 (32.9%)	1,369 (37.0%)	3,750 (34.5%)	5,122 (31.0%)
Other insurance	5,295 (17.0%)	508 (13.7%)	1,816 (16.7%)	2,971 (18.0%)
Not insured	1,588 (5.1%)	161 (4.4%)	532 (4.9%)	895 (5.4%)
Unknown	498 (1.6%)	77 (2.1%)	163 (1.5%)	258 (1.6%)
	Regional median income (*n*, %)			
<$63,000	22,663 (72.9%)	2,618 (70.7%)	7,740 (71.2%)	12,305 (74.4%)
$63,000+	7,912 (25.4%)	940 (25.4%)	2,831 (26.0%)	4,141 (25.1%)
Unknown	532 (1.7%)	143 (3.9%)	305 (2.8%)	84 (0.5%)

### 3.1 Anticancer therapy utilization for HCC 2004–2014

**FIGURE 1:**
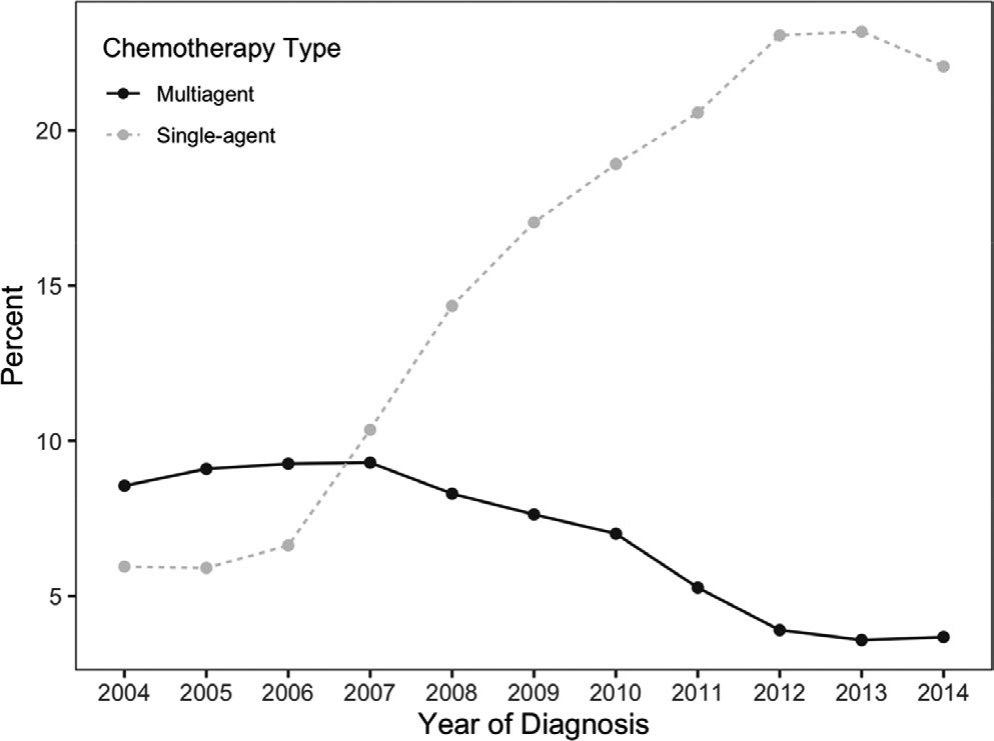
Overall utilization of anticancer therapy for aHCC

**FIGURE 2:**
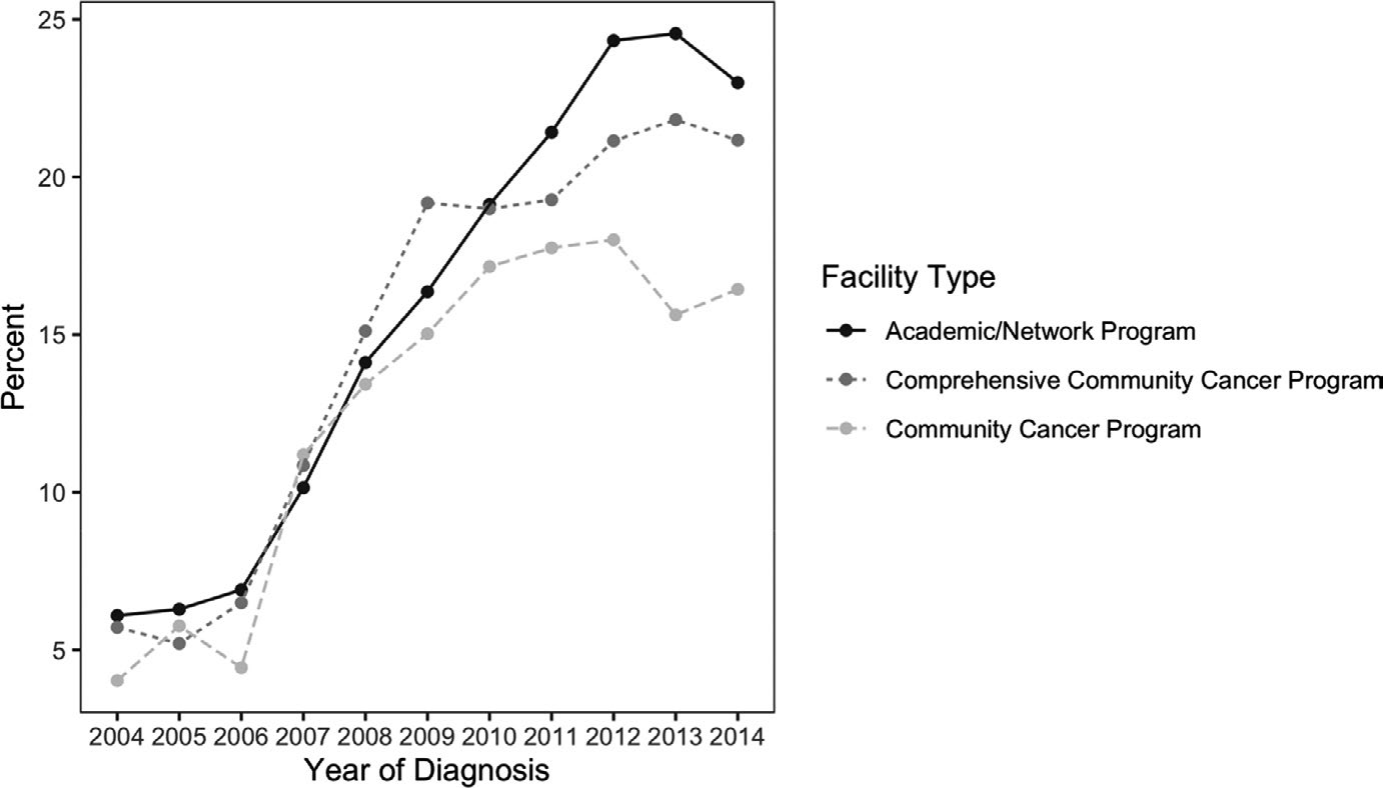
Utilization of single agent anticancer therapy by facility type

The use of anticancer therapy for aHCC changed significantly from 2004 to 2014. In the pre‐sorafenib period of 2004–2006, use of anticancer therapy for aHCC was relative stable at 15.1%. Among those receiving anticancer therapy, approximately three‐fifths of patients (40.1%) received SAACT. Soon after sorafenib approval, overall ACT utilization for aHCC increased significantly to approximately 23.2%. For those receiving ACT in the early sorafenib period of 2007–2010, 66.0% of patients received SAACT, and by the late sorafenib period of 2011–2014, 26.3% of all aHCC patients received ACT with 84.5% receiving SAACT (Figure 1). The overall use of multi‐agent ACT diminished to under five percent, which represents 15.5% of all patients receiving anticancer therapy. The increase in SAACT is noted to rise in 2007 to 52.7% from 41.7% the previous year. The use of SAACT was similar during the pre‐sorafenib period among the three facility types analyzed (academic/network, comprehensive community, and community centers) at 6.2%. This utilization increases to 15.2% in a relatively similar trend among the three facility types during the early sorafenib period. In the late sorafenib period, SAACT use continues to rise to an average of 22.2%, but we see a difference in utilization with academic/network centers having the highest use at 23.3% compared to comprehensive community centers (20.9%), and community centers (17.0%). (Figure 2).

### 3.2 Median Overall Survival with Anticancer therapy

**FIGURE 3:**
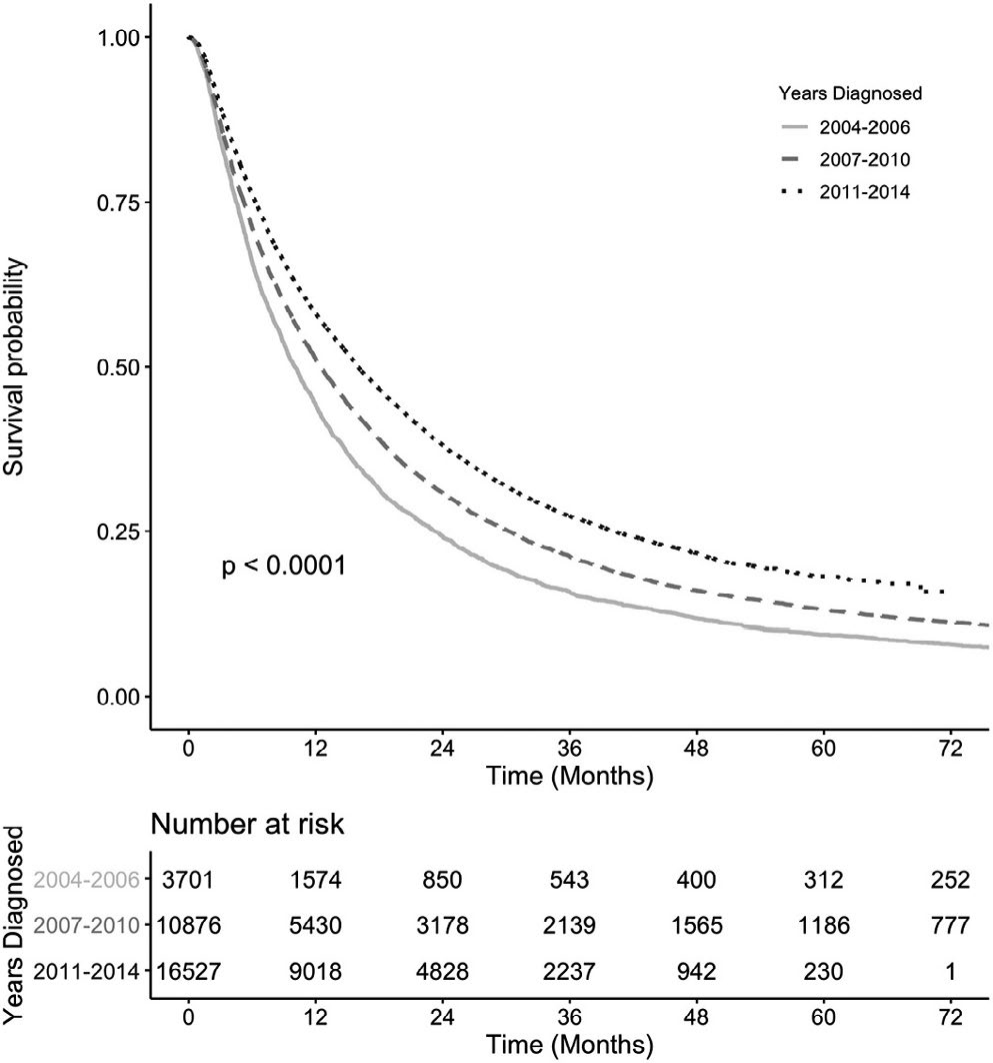
Kaplan‐Meier survival curves of aHCC patients treated with anticancer therapy by time period

**FIGURE 4:**
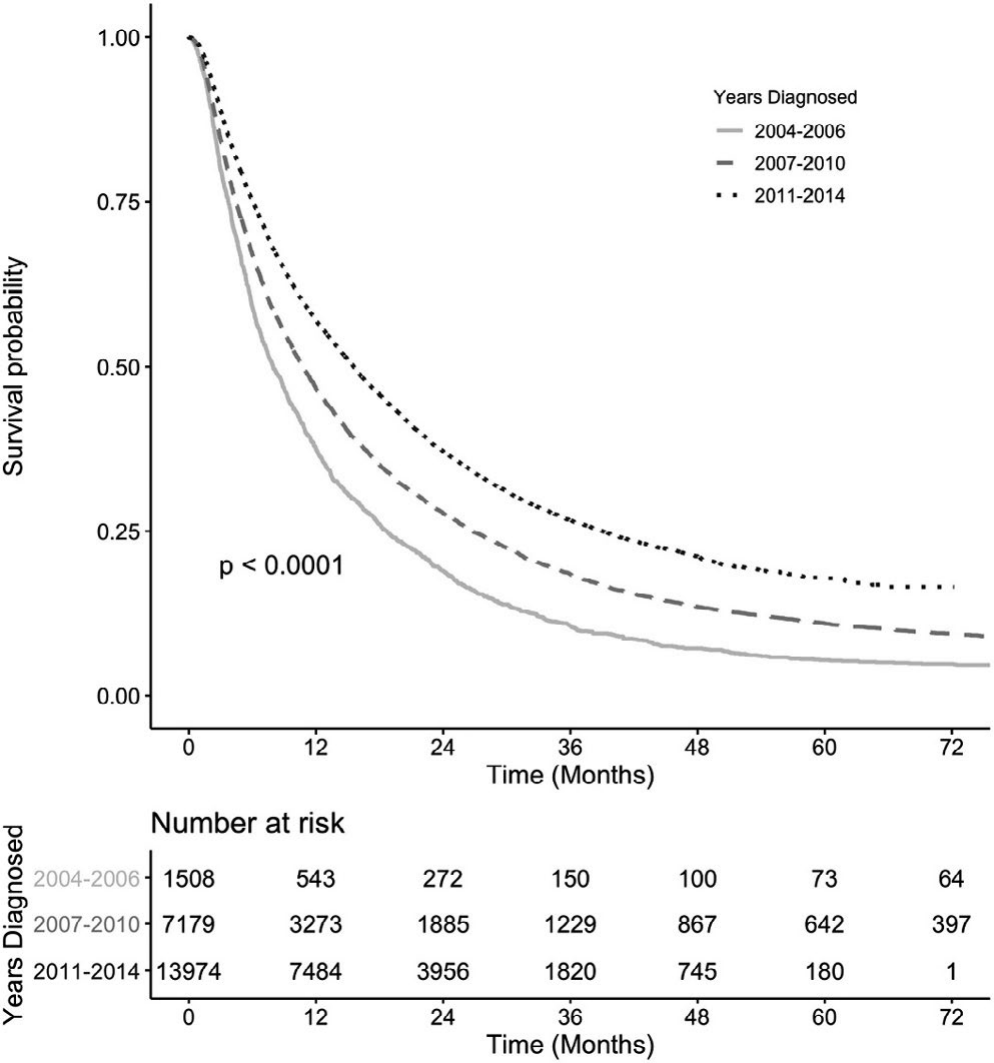
Kaplan‐Meier survival curves of aHCC patients treated with single agent anticancer therapy by time period

Analysis of the NCDB among patients with aHCC and treated only with ACT, the median overall survival (mOS) improved from 10.0 m (95% CI: 9.5–10.6) in the pre‐sorafenib period and to 12.5 m (95% CI: 12.0–12.9) in the early sorafenib period to eventually 16.0 m (95% CI: 15.6–16.4, *p* < 0.001) in the late sorafenib period (Figure 3). A similar trend was found for aHCC patients only receiving SAACT: 8.0 m (7.4–8.8) to 10.7 m (10.4–11.2) to 15.6 m (15.2–16.0), respectively (Figure 4). To explore the impact of facility type, we analyzed the survival of aHCC patients treated with anticancer therapy only. We found significant difference in survival at academic/network cancer centers as compared to comprehensive community and community cancer centers among all the three time frames (Table 2). The difference is most pronounced in the late sorafenib period from 2011–2014 with a mOS of 19.9 m (19.2–20.5) at academic/network cancer centers as compared to a mOS of 11.3 m (10.6–12.0) at comprehensive community cancer centers and a mOS of 8.1 m (7.5– 9.6) at community cancer centers (Table 2). This trend persisted when analyzing aHCC patients receiving SAACT: 18.1 m (17.5–18.8) vs. 11.2 m (10.5–12.0) vs. 7.7 m (7.1–9.1), respectively (Table 2). We also identified that aHCC patients with no insurance have a worse survival (HR 1.11, 95% CI: 1.06–1.16) as compared to those with insurance and similarly, those patients with estimated household annual income of <$63 k (HR 1.09, 1.05 – 1.13) had worse outcomes as compared with an estimated household annual income of ≥$63 k.

**TABLE 2:** Median overall survival of aHCC patients among facility type, by anticancer therapy type.

Facility type	ACT			SAACT		
	2004–2006	2007–2010	2011–2014	2004–2006	2007–2010	2011–2014
Academic & Network	11.9 m	15.6 m	19.9 m	8.8 m	12.9 m	18.1 m
	(11.0–12.5)	(15.0–16.3)	(19.2–20.5)	(7.9–9.7)	(12.3–13.6)	(17.5–18.8)
Comprehensive community	7.9 m	8.7 m	11.3 m	6.9 m	8.1 m	11.2 m
	(7.1–8.8)	(8.3–9.4)	(10.6–12)	(6.2–8.0)	(7.5–8.7)	(10.5–12)
Community	6.4 m	6.0 m	8.1 m	6.3 m	5.6 m	7.7 m
	(5.1–8.6)	(5.4–6.7)	(7.5–9.6)	(4.8–10.6)	(5.1–6.1)	(7.1–9.1)

aHCC – advanced hepatocellular carcinoma; ACT ‐ anticancer therapy; SAACT – single agent anticancer therapy

## 4 DISCUSSION

The use of targeted therapies has dramatically improved the overall survival of patients with aHCC in the United States. Because sorafenib was the only FDA‐approved therapy for aHCC for a decade, we had a unique opportunity to assess its adoption and impact on patient outcomes through the NCDB. We have confirmed increased utilization of SAACT with only a minority of patients being treated with multiagent ACT at the end of the time frame analyzed. Additionally, we found a trend toward improved median survival among patients with aHCC. Our study also indicates that the facility type appears to impact mOS as well as the socioeconomic factors of insurance and income.

This is the first study to document adoption of a newly FDA‐approved oral anticancer therapy for the treatment of aHCC in the United States. This is primarily due to the lack of any effective systemic therapies for patients with aHCC until 2007. Interestingly, we note that the use of SAACT begins to rise in 2007 by all facilities types despite the fact that the FDA approval did not occur until November 2007, late in the year. This would indicate that oncologists were already aware of the potential benefits of sorafenib for aHCC. The phase II study was published in 2006 and the results of the phase III study were presented at the ASCO Annual Meeting in 2007.^13^ However, in the late sorafenib period of 2012–2014, we identify a lower utilization of sorafenib among community cancer centers than in academic/network cancer centers by over 40%. Reasons for this difference is not available through NCDB and likely deserves further research to identify the cause of these differences.

Adoption of new standards of care is important so that patients may benefit from emerging clinical data, but only a few studies have reported on adoption of new systemic anticancer therapy. A study in advanced colorectal cancer found increased use of irinotecan and oxaliplatin chemotherapy for older patients and those with comorbidities from 1995 to 2009.^14^ In ovarian cancer, Melamed et al., found that regions that adopted neoadjuvant chemotherapy in stage 3C and 4 ovarian cancer patients led to a significant improvement in mortality rates as compared to regions that had no change in clinical practice.^15^ This adoption has also been found with oral anticancer therapy. One of the earliest examples is the use of oral anticancer therapy for the treatment of chronic myeloid leukemia with oral anticancer therapy use rising from 0% in 2000 to over 98% by 2005.^16^ In prostate cancer, Caram et al, also have noted significant increase in the use of oral anticancer therapy from 2013 to 2016 as these agents have become approved.^17^ In the current study, we have found not only early adoption of oral anticancer therapy prior to FDA approval but also have found a parallel improvement in clinical outcomes.

We find that the mOS has improved significantly with the increased utilization of SAACT from 8.0 m to 16.0 m from the pre‐sorafenib to late sorafenib periods. This improvement is significantly higher than the original publication, which showed a survival improvement of 2.8 m when compared to placebo. Clinical studies are often noted to include healthier patient populations and thus when applied to the general population, the clinical benefits may not be as significant. However, these data indicate that the survival difference noted in the SHARP studies was indeed applicable to the general population in the US and exceeded these results. A confounding factor is the general improvement in survival over time due to the improvements in supportive care and treatment of liver cirrhosis, including recently approved treatments for hepatitis C virus.

We also found a significant difference in mOS between facility types. Median overall survival in academic/network cancer centers was 11.9 m in pre‐sorafenib period and improved to 19.9 m in late sorafenib period whereas it was 6.4 m and 8.1 m at community centers over the same time periods for aHCC patients receiving ACT. Surprisingly, the relative improvement in mOS between these facility types was not similar – 67.2% vs. 26.6%, respectively. As a reference, the SHARP study showed a 35.4% improvement in survival as compared to placebo. Studies have reported a difference in overall survival in gastrointestinal cancers among facility type and these have focused on specific interventions, primarily surgery.^18^, ^19^, ^20^, ^21^, ^22^, ^23^ These differences in survival have been attributed due to academic centers having a higher volume of cases and thus have increased experience, knowledge, and resources in regard to the care of cancer patients and as it relates to complex procedures such as surgery. In contrast, this study focuses on the use of SAACT, and primarily the use of sorafenib, an oral anticancer therapy, which would presumably would be exactly the same medicine at all centers. Sorafenib was first approved for renal cell cancer in December 2005 and thus the relative experience of oncologists with sorafenib should be similar. Initially, we hypothesized the differences in survival among facility types was due to the early adoption of sorafenib by academic/network cancer centers. However, we found generally similar rates of adoption in the early sorafenib period. Only in the late sorafenib period did we find significant differences in SAACT utilization between facility types, specifically 37% higher in utilization of SAACT among academic/integrated centers as compared to community centers. Thus, there are likely other potential reasons for the large survival differences found between facility types. We did find a higher proportion of older patients >70 and T3/4 cancers among community centers as compared to academic/integrated centers: 33.2% vs 23.7% and 66.6% vs 40.5%, respectively. In contrast, academic/integrated centers had a higher proportion of patients with co‐morbidities ‐ Charlson score ≥2 (24.3% vs 18.6%). However, the multivariable survival model adjusted for these factors, and still found a significant difference in survival. Other potential contributing factors could include that clinicians at an academic/integrated center having more experience with managing aHCC and having more supportive care services to help address the symptoms associated with cirrhosis, aHCC, and sorafenib treatment. The study by Basch et al, demonstrates the importance of systematic symptom assessment and treatment for advanced cancer patients and that improvements in survival are possible.^24^

Worse outcomes for cancer patients have been associated with low socioeconomic factors, and similarly we found that having no insurance status and a household annual income <$63 K are associated with worse survival.^25^ This is consistent with most studies in HCC. Wang et al., used the SEER database from 2007–2012 and found that those HCC patients with Medicaid or no insurance were less likely to receive treatment and specifically those with no insurance had a worse survival (HR 1.39, 1.29–1.50; *p* < 0.001).^26^ Similarly, HCC patients with private insurance appear to have better survival outcomes than those with other types of insurance.^27^, ^28^ An earlier paper by Zaydfudim et al., also reported improved survival with private insurance, but noted that no difference in anticancer therapy utilization was found among the different insurance types.^29^ However, this analysis was conducted from 2004 to 2006, prior to the approval of Sorafenib. A more recent study has found that those with a low socioeconomic status were less likely to be prescribed sorafenib.^30^ Oral anticancer therapy may lead to special issues in obtaining the medication as compared to intravenous chemotherapy provided at an infusion center because of the co‐pay associated with oral prescription medications. Without insurance, the cost of sorafenib is approximately $5,000–8,000 per month with the cost rising from 2007 to 2012 and out of reach for most of the US cancer population.^31^ Even with Medicare, the annual out‐of‐pocket costs is estimated to be $5,063 in 2020.^32^ These issues may lead to a delay in seeking health care but also a delay in obtaining the appropriate treatment. Future research should consider examining if safety net hospitals associated with academic institutions that serve low socioeconomic populations have similar outcomes to those in the community. These healthcare disparities are a consistent problem and more work needs to be done to narrow this gap.

## 5 LIMITATIONS

Our study has limitations including use of retrospective data from the NCDB. These data are thought to only represent 60–70% of cancer care in the US. Retrospective data have limitations but these can help us understand trends in a large population over a certain period of time. In this study, we presumed that SAACT was sorafenib because it was the only FDA‐approved therapy during the study period evaluated. Regorafenib was approved in 2017 and thus likely not significantly represented in the time frame from 2004 to 2014. Thus, we feel confident that these data primarily represent sorafenib utilization. The NCDB does not provide some key information such as performance status nor the risk factors associated with HCC development, which are known prognostic factors. The Charlson/Deyo co‐morbidity index provides an indicator of health status, but does not fully replace the performance status. Additionally, the Charlson/Deyo scores for this cohort seemed low given 48% were reported to have a score of 0 despite the majority of patients having cirrhosis, which should have been scored at least as 1 for mild cirrhosis or 3 for moderate‐severe cirrhosis. This likely indicates that a significant proportion of participating CoC sites may not have appropriately scored the Charlson/Deyo index properly, and thus the dataset may underestimate the co‐morbidities of this cohort. We are unable to re‐assess this because the database does not provide individual medical diagnoses. The comprehensive community cancer centers and community cancer centers represent a smaller proportion of the total data set, 25.5% and 4.0%, respectively.

In summary, with improvements in the treatment of aHCC including systemic treatment options with newer targeted therapies and immunotherapy such as atezoluzimab and bevacizumab, patients with aHCC will continue to have improvements in survival.^33^ As these new treatment options are approved, more research is needed to understand the adoption of these new therapies and the significant differences in survival found between facility types. Additionally efforts are needed on how to provide support for patients with no insurance and lower income so that the clinical benefits are equally experienced by patients regardless of facility type and socioeconomic status.

## CONFLICTS OF INTEREST

The authors have declared no conflicts of interest.

## AUTHOR CONTRIBUTIONS

Aman Opneja ‐ conceptualization, formal analysis, investigation, methodology, writing ‐ original draft, and writing ‐ review and editing. Gino Cioffi ‐ conceptualization, formal analysis, investigation, methodology, writing ‐ original draft, and writing ‐ review and editing. Asrar Alahmadi ‐ formal analysis, writing ‐ original draft, and writing ‐ review and editing. Nelroy Jones ‐ formal analysis, writing ‐ original draft, and writing ‐ review and editing. Tin‐Yun Tang ‐ writing ‐ review and editing. Nirav Patil ‐ formal analysis, methodology, writing ‐ original draft, and writing ‐ review and editing. David L. Bajor ‐ writing ‐ review and editing. Joel N. Saltzman ‐ writing ‐ review and editing. Amr Mohamed ‐ writing ‐ review and editing. Ankit Mangla ‐ writing ‐ review and editing. Jill Barnholtz‐Sloan ‐ conceptualization, formal analysis, investigation, methodology, writing ‐ original draft, and writing ‐ review and editing. Richard T. Lee ‐ conceptualization, formal analysis, funding acquisition, investigation, methodology, project administration, resources, supervision, writing ‐ original draft, and writing ‐ review and editing.

## Data Availability

The data that support the findings of this study are available from the American College of Surgeons – Commission on Cancer. Restrictions apply to the availability of these data, which were used under license for this study. Data are available from the authors with the permission of the American College of Surgeons – Commission on Cancer. The data that support the findings of this study are available on request from the corresponding author. The data are not publicly available due to privacy or ethical restrictions.
